# The RNA-binding motif 45 (RBM45) protein accumulates in inclusion bodies in amyotrophic lateral sclerosis (ALS) and frontotemporal lobar degeneration with TDP-43 inclusions (FTLD-TDP) patients

**DOI:** 10.1007/s00401-012-1045-x

**Published:** 2012-09-21

**Authors:** Mahlon Collins, David Riascos, Tina Kovalik, Jiyan An, Kelly Krupa, Kristin Krupa, Brian L. Hood, Thomas P. Conrads, Alan E. Renton, Bryan J. Traynor, Robert Bowser

**Affiliations:** 1 Departments of Neurobiology and Pathology, University of Pittsburgh, Pittsburgh, PA USA; 2 Women’s Health Integrated Research Center at Inova Health System, Annandale, VA USA; 3 Neuromuscular Diseases Research Unit, Laboratory of Neurogenetics, National Institute on Aging, National Institutes of Health, Bethesda, MD USA; 4 Division of Neurology, Barrow Neurological Institute, NRC 4th Floor, 350 W Thomas Road, Phoenix, AZ 85013 USA

**Keywords:** Amyotrophic lateral sclerosis, Frontotemporal lobar degeneration, TDP-43, RNA-binding protein, RBM45, C9ORF72

## Abstract

RNA-binding protein pathology now represents one of the best characterized pathologic features of amyotrophic lateral sclerosis (ALS) and frontotemporal lobar degeneration patients with TDP-43 or FUS pathology (FTLD-TDP and FTLD-FUS). Using liquid chromatography tandem mass spectrometry, we identified altered levels of the RNA-binding motif 45 (RBM45) protein in the cerebrospinal fluid (CSF) of ALS patients. This protein contains sequence similarities to TAR DNA-binding protein 43 (TDP-43) and fused-in-sarcoma (FUS) that are contained in cytoplasmic inclusions of ALS and FTLD-TDP or FTLD-FUS patients. To further characterize RBM45, we first verified the presence of RBM45 in CSF and spinal cord tissue extracts of ALS patients by immunoblot. We next used immunohistochemistry to examine the subcellular distribution of RBM45 and observed in a punctate staining pattern within nuclei of neurons and glia in the brain and spinal cord. We also detected RBM45 cytoplasmic inclusions in 91 % of ALS, 100 % of FTLD-TDP and 75 % of Alzheimer’s disease (AD) cases. The most extensive RBM45 pathology was observed in patients that harbor the *C9ORF72* hexanucleotide repeat expansion. These RBM45 inclusions were observed in spinal cord motor neurons, glia and neurons of the dentate gyrus. By confocal microscopy, RBM45 co-localizes with ubiquitin and TDP-43 in inclusion bodies. In neurons containing RBM45 cytoplasmic inclusions we often detected the protein in a punctate pattern within the nucleus that lacked either TDP-43 or ubiquitin. We identified RBM45 using a proteomic screen of CSF from ALS and control subjects for candidate biomarkers, and link this RNA-binding protein to inclusion pathology in ALS, FTLD-TDP and AD.

**Electronic supplementary material:**

The online version of this article (doi:10.1007/s00401-012-1045-x) contains supplementary material, which is available to authorized users.

## Introduction

Amyotrophic lateral sclerosis (ALS) is the most frequent adult-onset motor neuron disease and is characterized by a degeneration of motor neurons, leading to progressive muscle weakening and a typical life expectancy of 2–5 years after disease onset [4]. Frontotemporal lobar degeneration (FTLD) is the second most frequent cause of dementia and is a clinically diverse syndrome, with phenotypes including behavioral changes, semantic dementia and progressive non-fluent aphasia, and characterized by cellular inclusions containing tau (FTLD-tau), TAR DNA-binding protein 43 (TDP-43: FTLD-TDP) or fused-in-sarcoma (FUS: FTLD-FUS) [25, 28].

The presence of cytoplasmic inclusions positive for ubiquitin in degenerating neurons is a pathological hallmark of ALS and FTLD [15, 19]. The observation that some ALS patients develop cognitive deficits with prominent frontal lobe features, and that the associated neuropathology resembles that of FTLD led to the idea that ALS and FTLD might be related [21, 33]. An intronic hexanucleotide repeat expansion in the *C9ORF72* gene (GGGGCC) has recently been shown to be the genetic cause of chromosome 9p21-linked ALS-FTLD, and accounts for 30–40 % of familial ALS and a similar portion of familial FTLD, further linking these two neurodegenerative disorders [8, 38]. RNA generated from genomic non-coding repeat expansions may disrupt normal RNA metabolism by sequestering RNAs and proteins involved in other transcription/translation events [46].

TDP-43 and FUS have been identified as components of ubiquitinated inclusions occurring in ALS patients without Cu/Zn superoxide dismutase mutations and in FTLD patients [23, 25]. Both TDP-43 and FUS are primarily located in the nucleus of cells, but mislocalize and form neuronal and glial inclusions in ALS, FTLD-TDP and FTLD-FUS [3, 17, 35]. Mutations in *TDP*-*43* and *FUS* have been identified as a genetic cause in approximately 4 % of familial ALS and in rare cases of FTLD [27]. Both TDP-43 and FUS bind numerous RNAs (reviewed in [17, 42]) and are abnormally processed in ALS [47], linking altered RNA metabolism to ALS, FTLD-TDP and FTLD-FUS [42].

During an unbiased mass spectrometry-based proteomic analysis of cerebrospinal fluid (CSF) from ALS and control subjects, we detected an increase in the RNA-binding motif 45 (RBM45) protein in the CSF of ALS patients. This protein is expressed at highest levels in the brain [44], and has been suggested to be up-regulated in animal models of spinal cord injury and nerve degeneration [32]. Furthermore, RNA recognition motifs are conserved between RBM45, TDP-43 and FUS. Therefore, we sought to further characterize RBM45 expression and distribution in the brain and spinal cord of ALS, FTLD-TDP and control subjects.

RBM45 protein was detected in the CSF and central nervous tissue of ALS and control subjects. We observed RBM45 in a punctate pattern predominately in the nucleus of neurons and glia in the hippocampus and spinal cord of control subjects. In ALS patients, RBM45 was also contained in cytoplasmic inclusions in motor neurons that were immunoreactive for TDP-43 and ubiquitin. RBM45 was evident within cytoplasmic inclusions in 100 % of FTLD-TDP and 75 % of AD patients. In contrast to TDP-43, we also detected RBM45 in the nucleus of neurons containing cytoplasmic inclusions. Finally, the most abundant RBM45 pathology was observed in ALS patients that harbor the hexanucleotide repeat expansion of the *C9ORF72* gene. Thus, RBM45 represents a new RNA-binding protein located in cytoplasmic inclusions typical of ALS and FTLD-TDP patients.

## Materials and methods

### Tissue samples

ALS and control post-mortem fixed and frozen tissue was obtained from the University of Pittsburgh Brain Bank and the Center for ALS Research. Clinical diagnoses were made by board certified neuropathologists according to consensus criteria for each disease. All human tissues were obtained through a process that included written informed consent by the subjects’ next of kin. The acquisition process was evaluated by the University of Pittsburgh Institutional Review Board/University of Pittsburgh Committee for Oversight of Research Involving the Dead and determined to be exempt from review by the full committee. Subject demographics are listed in Table 1. The average age for each subject category was 59.7 ± 11.2 years for ALS, 60.2 ± 11.2 years for controls, 76.7 ± 9.9 years for frontotemporal lobar degeneration with TDP-43 inclusions (FTLD-TDP) and 78.2 ± 7.3 years for Alzheimer’s disease (AD) patients. The post-mortem interval for each subject group was 7.3 ± 4.6 h for ALS, 6.6 ± 5.0 h for controls, 9.0 ± 7.5 h for FTLD-TDP, and 4.5 ± 1.0 h for AD patients. While there was a statistically significant difference in age across the subject groups due to the more advanced age of the FTLD-TDP and AD cases (*p* = 0.01), there was no significant difference of post-mortem interval (*p* = 0.6). All FTLD-TDP cases either presented with motor neuron disease or developed motor neuron deficits and were best fit neuropathologic criteria for FTLD-TDP type B with TDP-43 inclusions in spinal cord motor neurons and frontal and/or temporal cortex [24]. All AD cases were Braak stage VI with frequent plaque pathology by CERAD criteria [30, 31]. Two of four AD cases had additional TDP-43 pathology in the hippocampus, as noted in Table 2.

**Table 1:** Subject demographics

Case ID	Group	Age	Gender	PMT	*C9ORF72* repeat expansion
1	ALS	40	M	6	No
2	ALS	68	M	4	Yes
3	ALS	43	M	6	Yes
4	ALS	45	M	7	No
5	ALS	62	M	7	No
6	ALS	72	F	9	No
7	ALS	69	M	5	No
8	ALS	50	F	7	No
9	ALS	60	F	4	No
10	ALS	59	M	4	No
11	ALS	77	F	4	No
12	ALS	79	F	4	No
13	ALS	52	F	21	No
14	ALS	45	M	7	No
15	ALS	65	M	5	No
16	ALS	66	M	18	No
17	ALS	53	F	12	No
18	ALS	73	F	6	No
19	ALS	59	M	5	No
20	ALS	70	F	4.5	No
21	FALS	55	M	14	No
22	FALS	63	F	4	Yes
23	FALS	48	F	5	No
24	FTLD-TDP	69	M	6	No
25	FTLD-TDP	67	M	24	No
26	FTLD-TDP	69	M	6	No
27	FTLD-TDP	85	M	3	No
28	FTLD-TDP	91	F	8	No
29	FTLD-TDP	79	M	7	No
30	AD	73	F	4	No
31	AD	71	M	4	No
32	AD	85	M	5	No
33	AD	84	F	5	No
34	CT	54	M	6	No
35	CT	53	F	4	No
36	CT	76	M	13	No
37	CT	57	M	2	No
38	CT	48	M	2	No
39	CT	58	F	5	No
40	CT	76	M	14	No

Subject case number, disease group, age, gender, PMT (post-mortem interval), and the presence or absence of the *C9ORF72* repeat expansion. All AD cases were Braak stage VI

*ALS* amyotrophic lateral sclerosis, *FALS* familial amyotrophic lateral sclerosis, *FTLD-TDP* frontotemporal lobar degeneration with TDP-43 inclusions, *AD* Alzheimer’s disease, *CT* non-neurological disease control

**Table 2:** Immunohistochemistry results for RBM45 and TDP-43

Case ID	Lumbar spinal cord				Hippocampus			
	RBM45 pathology		TDP-43 pathology		RBM45 pathology		TDP-43 pathology	
	NCI	GCI	NCI	GCI	NCI	GCI	NCI	GCI
1	++	++	++	++	NA	NA	NA	NA
2*	+++	++	+++	+	NA	NA	NA	NA
3*	+++	++	+++	+	NA	NA	NA	NA
4	++	++	+	+	NA	NA	NA	NA
5	+	+	+	+	NA	NA	NA	NA
6	+	++	++	+++	NA	NA	NA	NA
7	−	−	+	++	+	−	+	−
8	−	+	+	+	NA	NA	NA	NA
9	++	−	++	+++	NA	NA	NA	NA
10	++	+	++	++	+	−	+	−
11	++	+	+	+	NA	NA	NA	NA
12	+	+++	+	++	−	−	+	−
13	+	+	+	−	++	−	+	−
14	++	++	+	+	+	−	++	−
15	++	++	+	++	−	−	−	−
16	−	−	++	+	−	−	+	−
17	+	+	+	−	+++	−	++	−
18	−	−	+	+	NA	NA	NA	NA
19	+	−	+	+	NA	NA	NA	NA
20	++	++	++	++	NA	NA	NA	NA
21	−	+	+	−	NA	NA	NA	NA
22*	++	+	++	+	−	−	+	−
23	+	+	++	+++	NA	NA	NA	NA
24	+	+	+	+	++	−	++	−
25	+	−	+	+	−	−	+++	−
26	NA	NA	NA	NA	+	−	+++	−
27	NA	NA	NA	NA	+++	−	+++	−
28	NA	NA	NA	NA	+++	−	+++	−
29	NA	NA	NA	NA	+	−	++	−
30	NA	NA	NA	NA	+++	−	+++	−
31	NA	NA	NA	NA	++	−	++	−
32	NA	NA	NA	NA	−	−	−	−
33	NA	NA	NA	NA	++	−	+	−
34	−	−	−	−	−	−	−	−
35	−	−	−	−	−	−	−	−
36	−	−	−	−	NA	NA	NA	NA
37	−	−	−	−	NA	NA	NA	NA
38	−	−	−	−	−	−	−	−
39	−	−	−	−	−	−	−	−
40	−	−	−	−	−	−	−	−

Cytoplasmic inclusions were counted in at least three tissue sections from each region

(−), no inclusions; (+), 1–3 inclusions per section; (++), 4–9 inclusions per section; (+++), greater than 10 inclusions per section

*NCI* neuronal cytoplasmic inclusions, *GCI* glial cytoplasmic inclusions

* Denotes cases with *C9ORF72* repeat expansion

### CSF collection, processing and mass spectrometry-based proteomics

The study population comprised 90 sporadic ALS (SALS), 20 familial ALS (fALS), 20 multiple sclerosis (MS), 20 Alzheimer’s disease (AD), 10 lower motor neuron disease (LMND), 5 upper motor neuron disease (UMND) and 80 healthy control (HC) subjects. Revised El Escorial criteria were used to diagnose all ALS subjects [5], with 18 % diagnosed as definite ALS, 33 % probable ALS, 24 % probable/lab supported ALS and 25 % possible ALS. Healthy controls were typically spouses or family friends of the ALS patients. Of the FALS patients, three had SOD1 mutations. All LMND subjects exhibited only lower motor neuron symptoms at the time of the lumbar spinal tap. All UMND subjects exhibited only upper motor neuron symptoms at the time of the lumbar spinal tap. CSF samples (between 3 and 10 mL per subject) were obtained by lumbar puncture from subjects upon informed patient consent. All samples were spun at 3,000 rpm at 4 °C for 10 min to remove any cells and debris, aliquoted in small volumes and stored in low bind polypropylene tubes at −80 °C within 2 h from harvesting. Only CSF samples without visible blood were processed by centrifugation, and hemoglobin levels in all final CSF samples were measured by ELISA to eliminate those with evidence of significant levels of hemoglobin denoting blood contamination.

We generated 25 pooled samples (Table 3), each containing 200 μl of CSF from 10 subjects that were age and gender-matched. CSF samples were concentrated using Amicon Ultra 3 K columns (Millipore) and adjusted to a volume of 400 μl using a 4× solution of Agilent Buffer A. Abundant proteins were depleted using the Agilent Multi-Affinity Removal spin cartridge that removes six highly abundant proteins according to the manufacturer’s protocol. Depleted samples were buffer exchanged into 50-mM ammonium bicarbonate (NH_4_HCO_3_) by centrifugation using AmiconUltra 3 K columns to a final volume of 300 μl and protein concentrations were determined by BCA (Pierce).

**Table 3:** Subject groups used for mass spectrometry based proteomics

Group #	Subject type	Age (years)
1	SALS, limb onset	<40
2	SALS, limb onset	<40
3	SALS, limb onset	40–60
4	SALS, limb onset, taking riluzole	40–60
5	SALS, limb onset, taking riluzole	40–60
6	SALS, bulbar onset	40–60
7	SALS, limb onset	>60
8	SALS, limb onset, taking riluzole	>60
9	SALS, bulbar onset	>60
10	FALS	40–60
11	FALS	>60
12	Healthy controls	<40
13	Healthy controls	<40
14	Healthy controls	<40
15	Healthy controls	40–60
16	Healthy controls	40–60
17	Healthy controls	40–60
18	Healthy controls	>60
19	Healthy controls	>60
20	Multiple sclerosis	<40
21	Multiple sclerosis	40–70
22	Lower motor neuron disease (LMN)	
23	Upper motor neuron disease (UMN)	
24	Alzheimer’s disease	>60
25	Alzheimer’s disease	>60

Each group contains 100 μl of CSF from each of 10 individuals, comprising six males and four females

*SALS* sporadic ALS, *FALS* familial ALS

The samples were reduced with addition of 10-mM DTT at 56 °C for 30 min, alkylated in 55 mM iodoaceteamide in the dark for 30 min, and 3 μl of 1 % ProteasMAX (Promega) and Trypsin Gold (Promega) were added at a 1:20 ratio and digested at 37 °C for 9 h. Finally, 15.75 μl of 1 % TFA was added and the samples stored at −80 °C. All samples were then de-salted using PIERCE Pepclean C-18 spin columns, peptides eluted with 0.1 % TFA and 60 % ACN and dried by vacuum centrifugation.

Peptide digests were resuspended in 0.1 % TFA and analyzed in triplicate by nanoflow reversed-phase liquid chromatography (LC)-MS/MS using a Dionex Ultimate 3000 LC system (Dionex Corporation, Sunnyvale, CA) coupled online to a linear ion trap (LIT) mass spectrometer (LTQ, ThermoFisher Scientific, San Jose, CA). Separations were performed using 75-μm i.d. × 360 o.d. × 10 cm long fused silica capillary columns (Polymicro Technologies, Phoenix, AZ) that were slurry packed in house with 5 μm, 300 Å pore size C-18 silica-bonded stationary phase (Jupiter, Phenomenex, Torrance, CA). Following sample injection onto a C-18 trap column (Dionex), the column was washed for 3 min with mobile phase A (2 % acetonitrile, 0.1 % formic acid in water) at a flow rate of 30 μl/min. Peptides were eluted using a linear gradient of 0.3 % mobile phase B (0.1 % formic acid in acetonitrile)/min for 130 min, then to 95 % B in an additional 10 min, all at a constant flow rate of 0.20 μl/min. Column washing was performed at 95 % B for 20 min, after which the column was re-equilibrated in mobile phase A prior to subsequent injections. The LIT-MS was operated in a data-dependent MS/MS mode in which each full MS scan was followed by five MS/MS scans where the five most abundant peptide molecular ions are selected for collision-induced dissociation (CID), using a normalized collision energy of 30 %. Data were collected over a broad mass to charge (*m/z*) precursor ion selection scan range of 375–1,800, utilizing dynamic exclusion to minimize redundant selection of peptides previously selected for CID.

Tandem mass spectra were searched against a combined UniProt human protein database (7/09 release) from the European Bioinformatics Institute (http://www.ebi.ac.uk/integr8) using SEQUEST (ThermoFisher Scientific). For a fully tryptic peptide to be considered legitimately identified, it had to achieve stringent charge state and proteolytic cleavage-dependent cross-correlation (×corr) scores of 1.9 for [M + H]^1+^, 2.2 for [M + 2H]^2+^ and 3.5 for [M + 3H]^3+^, and a minimum delta correlation (ΔCn) of 0.08. In addition, peptides were searched for methionine oxidation with a mass addition of 15.9949 and serine, threonine and tyrosine phosphorylation with a mass addition of 79.9663. A false peptide discovery rate less than 2 % was determined by searching the primary tandem MS data using the same criteria against a decoy database wherein the protein sequences are reversed [10]. Results were further filtered using software developed in-house, and differences in protein abundance between the samples were derived by summing the total CID events that resulted in a positively identified peptide for a given protein accession across all samples (spectral counting) [20]. Statistical analysis across the sample groups using the number of peptides for each protein was performed by unpaired *t* test using GraphPad Prism 5.0 software (La Jolla, CA). Effect size of each protein was determined using the number of peptides for each group and comparing all ALS groups versus all other groups, using the equation:

$$ {\text{Effect size }} = \,\frac{{\left[ {\text{Mean\, of\, all\, ALS \,groups}} \right] - \left[ {\text{Mean\, of\, all\, other\, groups}} \right]}}{\text{standard \,deviation}} $$

where standard deviation represents the standard deviation of all non-ALS groups [13].

### Immunohistochemistry

Paraffin-embedded tissue sections of spinal cord from ALS (*n* = 23), FTLD-TDP (*n* = 2), and non-neurologic disease control (*n* = 7), and hippocampus from ALS (*n* = 9), FTLD-TDP (*n* = 6), Alzheimer’s disease (*n* = 4) and non-neurologic disease control (*n* = 5) cases were used for this study. All sections were deparaffinized, rehydrated and antigen retrieval performed using Target Antigen Retrieval Solution, pH 9.0 (DAKO) for 20 min in a steamer. After cooling to room temperature, non-specific binding sites were blocked using Super Block (Scytek) for 1 h. The following primary antibodies were used for immunohistochemistry: affinity-purified rabbit polyclonal anti-RBM45 generated to amino acids 1–130 (1:75; Sigma-Aldrich Prestige antibody HPA020448), custom-made affinity-purified rabbit monoclonal antibody to the C-terminal 15 amino acids of RBM45 (1:200 dilution; PI462476), affinity-purified rabbit polyclonal anti-RBM45 antibody AV41154 (Sigma-Aldrich), rabbit polyclonal anti-TDP43 (1:10,000; Proteintech) and mouse monoclonal anti-Ubiquitin antibody (1:1,000; Cell Signaling) with overnight incubation. After three washes, tissue sections were incubated for 1 h in the appropriate biotinylated IgG secondary antibodies (1:200; Vector Labs) diluted in Super Block. Slides were washed in PBS for 15 min and immunostaining visualized using the Vectastain Elite ABC reagent (Vector Labs) and Vector NovaRED peroxidase substrate kit (Vector Labs). Slides were counterstained with hematoxylin (Sigma Aldrich). Sections were visualized using an Olympus BX40 light microscope and images acquired using a Nikon DS L2 digital camera.

Semi-quantitative assessment of RBM45 and TDP-43 cytoplasmic inclusions was performed on all coded sections of the lumbar spinal cord and hippocampus by three independent investigators. The following scoring system was used: (−) = none; (+) = 1–3 inclusions per section; (++) = 4–9 inclusions per section; (+++) = 10 or more inclusions per section.

Quantitative assessment of RBM45 and TDP43 pathology was performed on select spinal cord sections. The gray matter was morphologically identified for each lumbar spinal cord section on pictures at 1.25× magnifications using a Leica microscope and outlined using NIH ImageJ software. The area of the gray matter was calculated via the ROI (region of interest) tool of NIH ImageJ software (1 pixel = 1.276 μ). Then, counts of total motor neurons, neuronal and glia inclusions for both RBM45 and TDP-43 were established per slide and results reported as a proportion of gray matter area density for RBM45 and TDP-43 inclusions.

### Confocal microscopy

For confocal microscopy, 6-μm sections were deparaffinized, rehydrated, subjected to antigen retrieval, and blocked as above. Following blocking, the slides were incubated overnight in affinity-purified rabbit polyclonal anti-RBM45 primary antibody HPA020448 (1:75; Sigma Aldrich). For double-label experiments, mouse monoclonal anti-TDP43 antibody (1:100; Proteintech), mouse monoclonal anti-phosphorylated Tau AT100 antibody (1:100; Thermo Scientific), or mouse monoclonal anti-Ubiquitin antibody (1:100; Cell Signaling) was also included. Appropriate IgG secondary antibodies were used labeled with either Alexa 488 or Alexa 594 fluorophores. Tissue sections were washed 3 times for 10 min in PBS after all antibody incubations. Cell nuclei were stained using DAPI (1:1,000). Confocal images were acquired using a Zeiss LSM 710 confocal microscope.

Images were analyzed and quantified for RBM45 and TDP-43 pathology and co-localization using Zeiss Zen software (version 2009) using 0.5-μm Z-stack sections. We determined the percent overlap between RBM45 and TDP-43 in at least 50 cells per subject group.

### Immunoblot and tissue homogenization

CSF samples from non-neurologic disease controls (*n* = 9) and ALS (*n* = 9) were analyzed for immunoblot. The average age was 54.7 ± 11.9 years for the controls and 49 ± 6.4 years for the ALS patients, with no statistically significant difference between the groups (*p* = 0.30). All subjects were male. All ALS patients were limb onset with an average time from symptom onset to lumbar tap of 782 days. Equal amounts of CSF protein (10 μg) from each subject was mixed in SDS sample buffer and heated for 10 min at 90 °C. Samples used for CSF analysis were from living ALS patients or controls and therefore these subjects are not included in the neuropathologic analysis.

For generation of total tissue homogenates, lumbar spinal cord frozen tissue from controls (*n* = 2) and ALS cases (*n* = 4) were homogenized in lysis buffer containing 25 mmol/l HEPES (pH 7.4), 50 mmol/l NaCl, protease inhibitor cocktail II (Sigma), and 1 % Triton-X 100. The lysates were spun at 14,000 rpm for 5 min at 4 °C and the supernatant saved as the total cell lysate. Protein concentrations of all samples were determined by BCA assay (Thermo-Fisher Scientific, Waltham, MA).

All samples were fractionated by electrophoresis on 4–12 % NuPAGE Bis–Tris gels in 1× MOPS Running Buffer at 200 V for 50 min. Proteins were transferred to polyvinylidene difluoride nylon membranes (NEN Biolabs) at 100 V for 1 h at 4 °C and blocked in 5 % non-fat milk/TBS-T. The blots were probed individually with a primary antibody for RBM45 (Sigma, AV41154 that recognizes amino acids 216-267 of RBM45) at 1:2,000 or TDP-43 (rabbit polyclonal 10782-1-AP; ProteinTech Group Inc.) at 1:1,000 dilution overnight at 4 °C in 1 % milk/TBST. The epitope recognized by AV41154 is not similar to any sequences in TDP-43 or FUS and recognizes only one major band by western blot. The final reaction products were developed using SuperSignal West Pico Chemiluminescent Substrate for 5 min and the band intensities were within the linear range of detection. The integrated optical density of bands was measured using the NIH Image J software and statistical analysis performed by Student’s *t* test using GraphPad Prism 5.0 software.

### Repeat-primed PCR

100 ng of genomic DNA was used as template in a final volume of 28 μl containing 14 μl of FastStart PCR Master Mix (Roche Applied Science, Indianapolis, IN) and a final concentration of 0.18 mM 7-deaza-dGTP (New England Biolabs, Ipswich, MA), 1× Q-Solution (Qiagen), 0.7 μM reverse primer consisting of ~4 GGGGCC repeats with an anchor tail, 1.4 μM 6FAM-fluorescent labeled forward primer located 280 bp telomeric to the repeat sequence, and 1.4 μM anchor primer corresponding to the anchor tail of the reverse primer. A touchdown PCR cycling program was used where the annealing temperature was gradually lowered from 70 to 56 °C in 2 °C increments with a 3-min extension time for each cycle. Fragment length analysis was performed on an AB 3730xl genetic analyzer (Applied Biosystems, Foster City, CA) and data analyzed using GeneScan software (version 4, ABI). The repeat-primed PCR is designed so that the reverse primer binds at different points within the repeat expansion to produce multiple amplicons of incrementally larger size, producing a characteristic sawtooth pattern with a 6-bp periodicity.

## Results

### Proteomic analysis of CSF and identification of RBM45

We performed an unbiased mass spectrometry-based proteomic analysis of CSF samples from ALS, healthy controls, multiple sclerosis, Alzheimer’s disease, upper motor neuron disease and lower motor neuron disease patients to discover additional candidate biomarkers for ALS. A complete and detailed description of all the proteins and cellular pathways that appeared altered in ALS versus the various controls groups will appear elsewhere. However, from the top 400 proteins identified in the CSF, we detected peptides from 13 nucleic acid-binding proteins in the CSF, with many of them altered in ALS patients (Table 4). Seven exhibited statistically significant increased peptide counts in the CSF of all subjects when compared across all other groups (healthy control and disease controls). Of these, the RNA-binding protein motif 45 (RBM45) appeared in almost all ALS groups but only a few of the control groups and was most statistically significant and exhibited the largest effect size between ALS and all other groups. Six other RNA-binding proteins were detected in the CSF, two of which also had statistically significant alterations in ALS groups versus all other subject groups (RNA-binding protein 40 and nucleolar RNA helicase 2). Peptides to both TDP-43 and FUS were only detected in a few of the subject groups using this mass spectrometry-based proteomic method and therefore were not in the top 400 proteins detected in CSF.

**Table 4:** Peptide counts for nucleic acid-binding proteins in each subject group

Protein	UniProtKB	ALS1	ALS2	ALS3	ALS4	ALS5	ALS6	ALS7	ALS8	ALS9	FALS1	FALS2	HC1	HC2	HC3	HC4	HC5	HC6	HC7	HC8		MS1	MS2	UMND	LMND	AD1	AD2	*p* value	Effect size
RNA-binding protein 45	Q8IUH3	4	2	0	1	1	1	1	2	2	1	1	0	0	0	0	0	1	0	0	0		1	0	1	0	0	0.001	2.91
Ribonuclease T2	O00584	5	6	5	3	1	2	0	1	0	1	3	2	2	3	6	3	3	2	0	2		0	2	2	1	1	0.60	0.257
jun-B	P17275	3	2	2	1	1	0	4	1	3	1	1	2	2	0	1	2	2	0	1	0		1	1	0	0	1	0.06	0.963
RNA polymerase I	P17480	1	1	1	1	1	0	1	1	0	0	0	0	0	0	0	0	0	0	0	0		0	0	1	0	0	0.002	2.11
Transcription factor TBX3	O15119	2	2	1	2	1	1	1	2	1	1	0	0	0	0	0	0	1	0	0	0		1	1	1	2	1	0.007	1.19
RNA helicase DDX59	Q5T1V6	3	1	3	0	0	0	0	0	1	1	0	2	0	0	0	0	0	0	0	0		0	0	0	0	0	0.06	1.26
RNA-binding protein 40	Q96LT9	3	0	0	2	1	2	1	1	0	0	2	0	0	1	0	0	0	0	0	0		0	0	0	0	0	0.002	2.81
Nucleolar RNAhelicase 2	Q9NR30	2	4	1	3	1	2	2	1	1	1	2	0	1	2	0	0	1	0	0	1		1	0	1	0	0	0.001	2.02
RNA-binding protein Raly	Q9UKM9	2	1	0	2	1	1	5	2	2	2	3	2	8	1	1	1	0	1	2	1		0	0	0	1	0	0.39	0.30
ZFR2	Q9UPR6	2	1	0	0	1	1	0	1	0	0	0	2	1	0	0	1	1	0	0	0		1	0	1	0	0	0.87	0.07
MLXIPL	Q9NP71	3	2	3	7	4	2	3	3	6	3	3	5	4	3	2	4	4	1	2	0		0	0	2	1	2	0.043	0.84
RAD54-like protein	Q92698	4	0	0	0	2	0	0	2	0	0	0	0	0	0	0	0	0	0	0	0		0	0	0	0	1	0.08	2.45
SPO11	Q9Y5K1	4	2	5	3	1	2	2	1	3	1	1	1	2	1	1	0	0	0	3	0		0	0	0	1	0	0.002	1.76

Unique peptides to each protein detected in 9 sporadic ALS, 2 familial ALS (FALS), 8 healthy controls (HC), 2 multiple sclerosis (MS), 1 upper motor neuron disease (UMND), 1 lower motor neuron disease (LMND) and 2 Alzheimer’s disease (AD) subject groups as described in Table 3. Statistical analysis performed using Student’s *t* test comparing number of peptides in ALS subject groups versus number of peptides in all control groups, with *p* value <0.05 considered statistically significant. Effect size was determined as described in the “Materials and methods” using the number of peptides in each group and comparing ALS to all other groups

RBM45 is a 476 amino acid protein that exhibits structural similarities with TDP-43 and FUS (Supplemental Figure 1), two RNA-binding proteins contained in cytoplasmic inclusions of neurons and glia in ALS and FTLD patients. RBM45 contains three RNA recognition motifs (RRMs) whereas TDP-43 has two and FUS has one RRM. RBM45 contains a C-terminal nuclear localization sequence, similar to FUS, but lacks both the glycine-rich domains contained in both TDP-43 and FUS and a defined nuclear export sequence (Supplemental Fig. 1). RNA-binding protein 40 (Table 4) contains two RRM domains and will be further characterized in a future study. However, the gene is located on the Y chromosome and is believed to participate in spermatogenesis [22], thus making a direct link to ALS less obvious.

### RBM45 protein levels in the CSF and tissue extracts

**Fig. 1:**
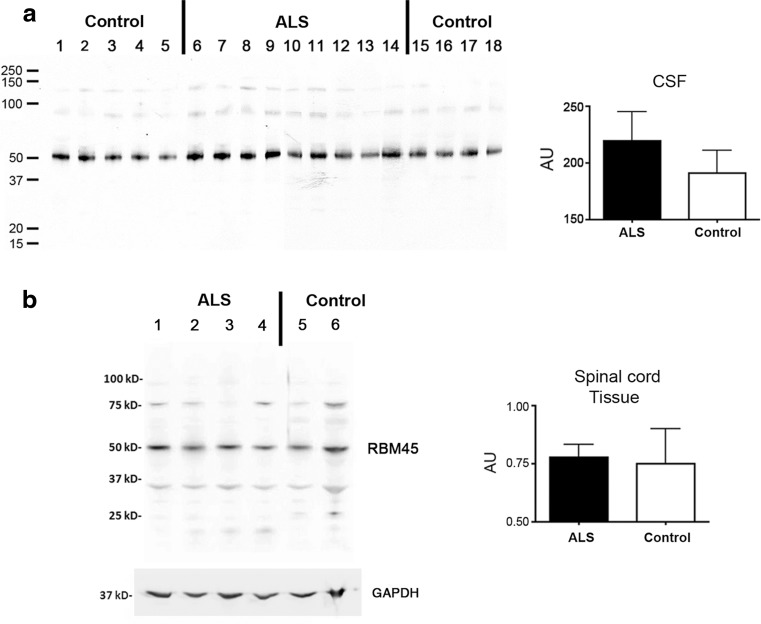
RBM45 immunoblot analysis. **a** Representative immunoblot comparing the level of RBM45 between 9 control and 9 ALS CSF samples. Equal amount of protein (10 μg) was loaded on each gel lane. *Right panel* is a densitometric analysis for all cases and comparison between ALS and controls. The difference between ALS and controls was not statistically significant by Mann–Whitney *t* test (*p* = 0.28). **b** Immuoblot analysis of lumbar spinal cord tissue extracts. The band representative of RBM45 is similar across all samples. Glyceraldehyde 3-phosphate dehydrogenase (GAPDH) levels were used as a loading control. The *right panel* is a densitometric analysis for all cases normalized to the level of GAPDH in each sample. No significant difference was seen between ALS and controls (*p* = 0.89). *AU* Arbitrary units

We first verified the presence of RBM45 in the CSF and spinal cord tissue extracts of ALS and control subjects by immunoblot. Using an affinity-purified antibody that recognizes amino acids 216–267 of RBM45 that lacks homology to TDP-43 or FUS, RBM45 protein was evident in all CSF samples, with a modest increase in ALS patients (Fig. 1a). We next prepared total protein extracts from spinal cord tissue of ALS and control subjects and detected similar levels of RBM45 protein in all samples (Fig. 1b). Therefore, we verified the presence of RBM45 in both CSF and spinal cord tissue, with a trend towards increased CSF levels in ALS patients.

### Distribution of RBM45 in control and ALS spinal cord

**Fig. 2:**
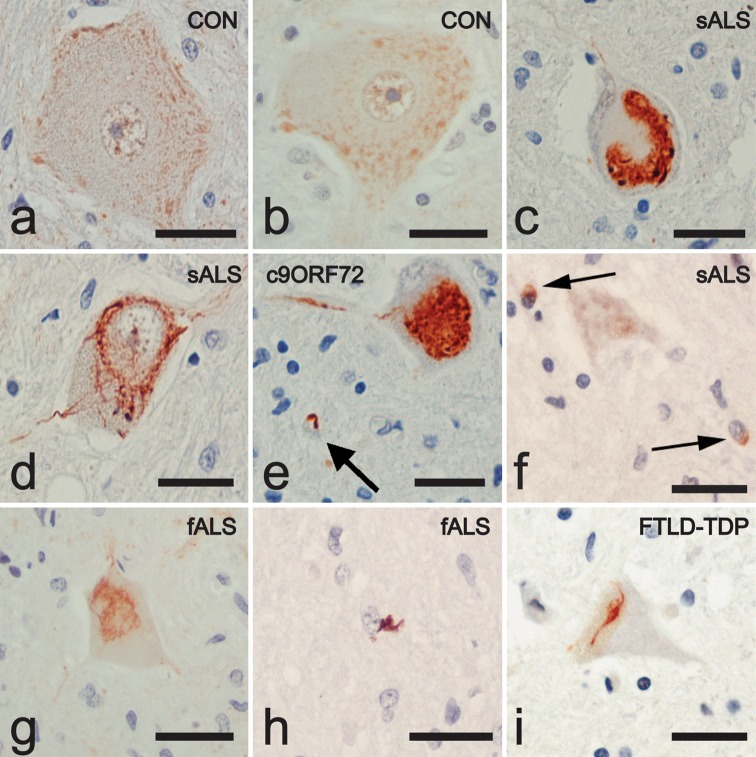
RBM45 distribution in spinal cord by light microscopy. Representative sections are shown from lumbar spinal cord sections from control, ALS and FTLD-TDP patients stained for RBM45 and counterstained with hematoxylin. **a**, **b** Motor neurons from control subjects show a punctate staining of the nucleus and cytoplasm. **c**–**e** Motor neurons from ALS patients, including a *C9ORF72* case in **e**, contain RBM45-positive inclusions with globular, skein-like and neuritic morphology. *Arrow* in **e** indicates a glial inclusion. **f** Two glial inclusions from a sporadic ALS patient are shown (*arrows*). **g** RBM45 positive inclusions were also detected in the motor neurons of non-SOD1, non-*C9ORF72* fALS cases. **h** Glial inclusions are also observed in fALS. **i** Spinal cord motor neuron of FTLD-TDP case containing skein-like RBM45 inclusion. All images are taken at ×40 magnifications. *Scale bars* equal 30 μm. *Panels* represent the following case numbers in Table 1: **a** 37, **b** 39, **c** 2, **d** 2, **e** 3, **f** 13, **g** 23, **h** 21, **i** 24

We next examined the cell type-specific expression patterns and subcellular localization of RBM45 in ALS and control subjects using immunohistochemistry. Non-neurologic disease control subjects exhibited a punctate staining pattern for RBM45 in the nucleus and cytoplasm of motor neurons in the lumbar spinal cord, with limited staining of nuclei within glial cells (Fig. 2a, b). Examination of sporadic and non-SOD1 familial ALS spinal cord tissue revealed RBM45-positive inclusion pathology bearing a striking resemblance to that seen with TDP-43 or FUS in ALS motor neurons (Fig. 2c–e). Several distinct morphologies were observed, including skein-like (Fig. 2d), globular (Fig. 2c, e), and neuritic (Fig. 2d, e) inclusions. Prior studies have demonstrated a clearance of TDP-43 from the nucleus of neurons that harbor cytoplasmic inclusions [23, 36]. However, we observed motor neurons with cytoplasmic RBM45 inclusions that retained RBM45 in a speckled or diffuse staining pattern in the nucleus (Fig. 2d). In addition, we were able to detect glial inclusions that stained positive for RBM45 (Fig. 2e, f). We also detected RBM45 inclusions in the spinal cord of familial ALS and FTLD-TDP cases (Fig. 2g–i). A semi-quantitative assessment of RBM45 and TDP-43 pathology is shown in Table 2. Within the spinal cord, we observed nuclear cytoplasmic RBM45 inclusions in 78 % of ALS patients (*n* = 18 of 23 cases). RBM45 glial cytoplasmic inclusions were observed in the spinal cord from 74 % of ALS patients (*n* = 17 of 23 cases). Two cases displayed RBM45 glial inclusions but no motor neuron inclusions (cases 18 and 21). Conversely, no RBM45-containing inclusions were observed in either cell type in the spinal cord of control subjects. From these data, there appeared to be a relationship between the RBM45 and TDP-43 pathology that was further examined by double-label confocal microscopy as described below.

The anti-RBM45 antibody used above recognizes amino acids 1–50 that contains a small region of the first RRM and therefore potential cross-reactivity with other proteins such as TDP-43 and FUS that contain these domains. However, a blocking peptide to this epitope completely eliminates immunoreactivity (Supplemental Figure 2), suggesting that this antibody does not also recognize similar epitopes in TDP-43 or FUS. In addition, RBM45 amino acids 1–50 exhibit little sequence identity to either TDP-43 or FUS. However, we also used another commercial antibody to RBM45 that recognizes amino acids 216–257 (lacks any RRM domain sequences) and a custom affinity-purified rabbit monoclonal antibody to the terminal 15 amino acids of RBM45 for immunohistochemistry. Both of these antibodies could detect RBM45 inclusions in either the spinal cord or hippocampus (Supplemental Figure 2).

Using a repeat-primed polymerase chain reaction (PCR) method [38], we determined that three ALS patients harbored *C9ORF72* repeat expansions, defined as having greater than 30 repeats (Table 1). This represented 10 % of our sporadic ALS and 33 % of our familial ALS population. Interestingly, these subjects exhibited the highest number of neuronal and glial cytoplasmic RBM45 inclusions (Table 2). This is similar to a recent study indicating that individuals with the *C9ORF72* repeat expansion had the highest number of p62 positive cytoplasmic inclusions in the lumbar spinal cord and hippocampus [7]. Within the lumbar spinal cord, ALS patients with the *C9ORF72* repeat expansion also exhibited the highest amount of TDP-43 inclusions (Table 2). We quantified the amount of RBM45 and TDP-43 inclusions within the lumbar spinal cord gray matter for 2–4 sections of each ALS case (see “Materials and methods”). *C9ORF72* positive cases contained on average 12.6 RBM45 neuronal inclusions per mm^2^ of gray matter whereas all other ALS cases contained 3.4 RBM45 neuronal inclusions per mm^2^ of gray matter. There was no difference in the number of RBM45 inclusions within glia in *C9ORF72* repeat expansion cases.

### RBM45 immunohistochemistry in the hippocampus

**Fig. 3:**
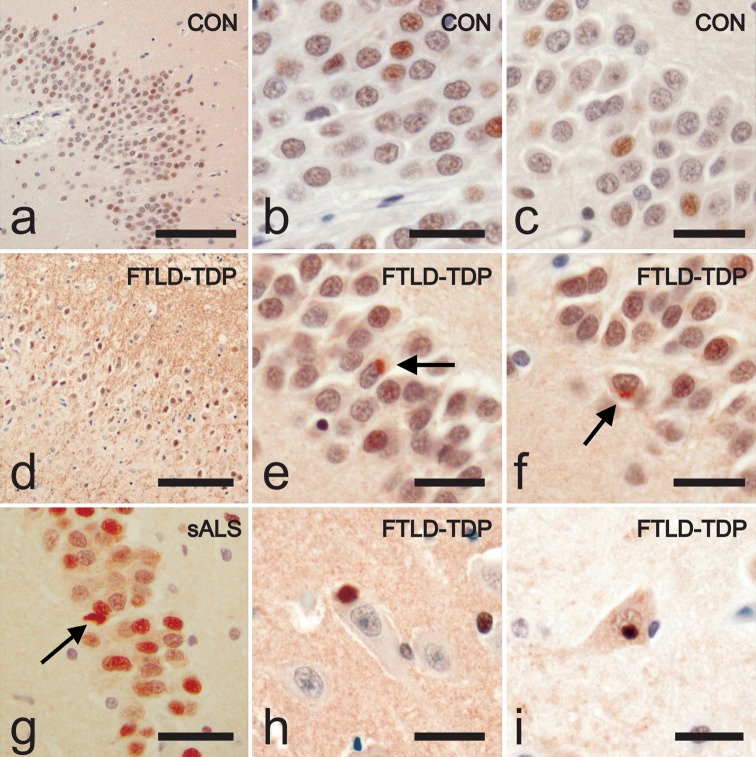
RBM45 localization in the hippocampus. Representative hippocampal sections from ALS, FTLD-TDP and control subjects immunostained for RBM45 and counterstained with hematoxylin. **a**–**c** RBM45 immunoreactivity in non-neurologic disease controls. RBM45 is located in the nucleus of dentate granule cells. **d**–**f** RBM45 cytoplasmic inclusions are observed in dentate granule cells in FTLD-TDP cases (*arrows* in **e** and **f**). **g** RBM45 pathology in the hippocampus of sporadic ALS. **h**, **i** Rare CA3 pyramidal neurons in FTLD-TDP cases contained RBM45 inclusions. For *panels* **a** and **d**, the magnification is ×10 and the scale bars denote 60 μm, while in all *other panels* the magnification is ×40 and *scale bars* indicate 20 μm. *Panels* represent the following case numbers in Table 1: **a** 38, **b** 40, **c** 39, **d** 29, **e** 27, **f** 26, **g** 17, **h** 29, **i** 24

**Fig. 4:**
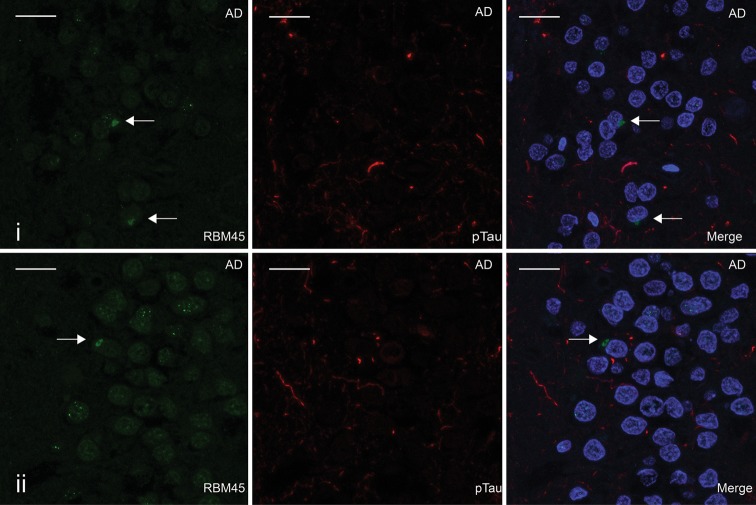
RBM45 and tau pathology do not overlap in AD cases. **a**, **b** Hippocampal sections from two AD cases stained for RBM45 (*green*) and phosphorylated tau (*red*) with nuclei (DAPI-*blue*) in the merged image. Abundant pTau pathology is seen in both cases, as well as RBM45 inclusions marked with *arrows*. No overlap of pTau pathology with RBM45 inclusions or speckled RBM45 nuclear staining is seen. *Scale bar* 20 μm. *Panels* represent the following case numbers in Table 1: **a** 30, **b** 33

Since TDP-43 pathology is also evident in the dentate of FTLD-TDP cases, we next evaluated the distribution of RBM45 in the hippocampus from FTLD-TDP, ALS and control subjects. Control subjects, including both non-neurologic controls and Alzheimer’s disease, exhibited a punctate or speckled RBM45 pattern within the nucleus of dentate granule cells (Fig. 3a–c). In FTLD-TDP patients there were RBM45 positive cytoplasmic inclusions within dentate granule cells (Fig. 3d–f). A speckled RBM45 immunoreactivity was still evident in the nucleus of dentate granule cells in FTLD-TDP cases. RBM45 inclusions were also detected in dentate granule neurons of ALS cases (Fig. 3g), as well as some hippocampal pyramidal neurons in ALS and FTLD-TDP patients (Fig. 3h, i). RBM45 dystrophic neurites were not detected in the hippocampus. Of the available hippocampal tissue, we observed RBM45 neuronal inclusions in 63 % of ALS cases (*n* = 5 of 8), with no glial inclusions in any case. While only six FTLD-TDP cases were available for this study, RBM45 pathology occurred in the hippocampus and/or spinal cord of all cases. RBM45 inclusions were also observed in the dentate gyrus and pyramidal neurons for three of four AD cases (Table 2). We used confocal microscopy to determine association of RBM45 with tau pathology in AD cases. RBM45 did not co-localize with phosphorylated tau (pTau) in the hippocampus of AD cases (Fig. 4). A speckled RBM45 pattern was also detected within the nuclei of dentate cells in AD cases (Fig. 4a, b). Finally, we detected no RBM45 pathology in the hippocampus of any non-neurologic disease control case.

### Co-localization of RBM45 with TDP-43

**Fig. 5:**
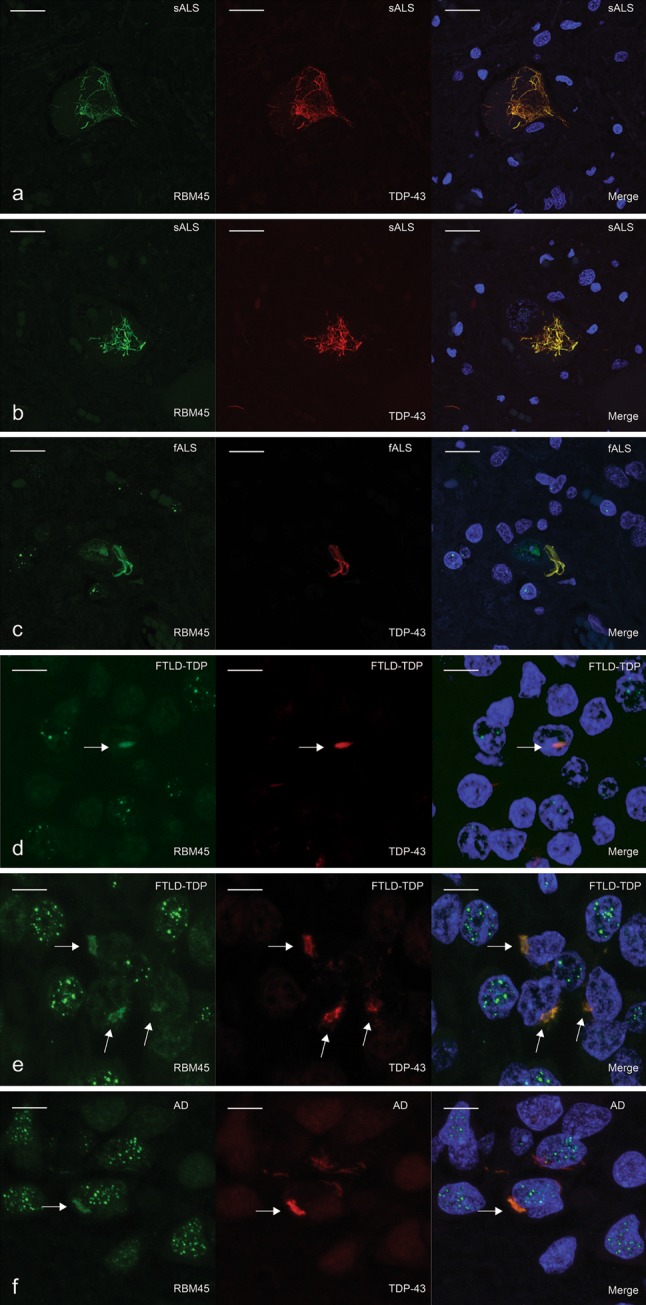
Double-label immunofluorescence for RBM45 (*green*) and TDP-43 (*red*) in the spinal cord and hippocampus. **a**–**c** RBM45 co-localization with TDP-43 positive inclusions in ALS spinal cord motor neurons. DAPI visualizes nuclei (*blue*) in the merged images. RBM45 also remained in the nucleus of a motor neuron with cytoplasmic TDP-43 inclusions (**c**). **d**–**f** RBM45 co-localization with TDP-43 inclusions in the dentate gyrus of FTLD-TDP (**d**, **e**) and AD (**f**) cases. *Arrows* denote co-localization in intranuclear (**d**) and cytoplasmic (**e**, **f**) inclusions. Speckled RBM45 nuclear stain is observed in *all panels* and is devoid of TDP-43. *Scale bars* denote 20 μm in *panels* **a**–**c** and 30 μm in *panels* **d**–**f**. *Each panel* represents the following case numbers in Table 1: **a** 2, **b** 3, **c** 7, **d** 24, **e** 28, **f** 30

We next examined the co-localization of RBM45 and TDP-43 pathology in ALS, FTLD-TDP and AD by double-label confocal microscopy, with DAPI to identify nuclei. We detected co-localization of RBM45 and TDP-43 within cytoplasmic inclusions of spinal cord motor neurons in ALS (Fig. 5a–c). We also noted ALS motor neurons with both nuclear RBM45 and co-localization in cytoplasmic TDP-43 inclusions (Fig. 5c). Interestingly, Fig. 5a, b represents ALS cases with the *C9ORF72* repeat expansion and exhibits reduced RBM45 nuclear staining when compared to ALS cases without the repeat expansion.

**Fig. 6:**
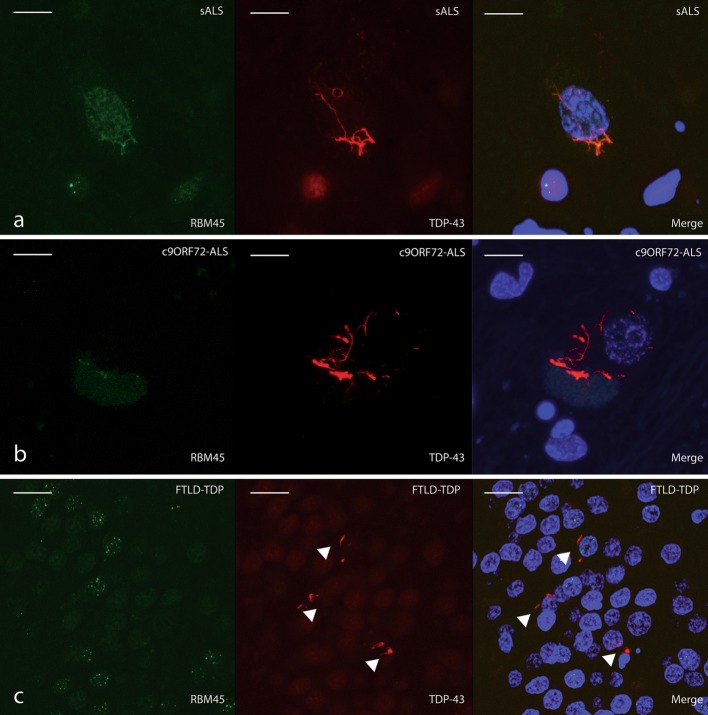
TDP-43 pathology can occur independent of RBM45 pathology in ALS and FTLD cases. RBM45 is labeled in *green*, TDP-43 denoted in *red* and DAPI (*blue*) visualizes nuclei in the merged images. **a** Motor neuron with nuclear RBM45 and a TDP-43 positive inclusion that labels poorly for RBM45. Several such inclusions were found throughout the lumbar spinal cord of sALS and fALS cases. **b** Motor neurons from *C9ORF72* ALS cases exhibited nuclear depletion of RBM45. RBM45 was not contained in the TDP-43 inclusions in this motor neuron. **c** Several TDP-43 positive, RBM45 negative inclusions are indicated by *arrowheads* in the dentate gyrus of an FTLD case. While no RBM45 positive inclusions were seen, the speckled nuclear staining pattern was observed in several adjacent cells. *Scale bar* 20 μm. *Panels* represent the following case numbers in Table 1: **a** 7, **b** 2, **c** 27

In the hippocampus of FTLD-TDP and AD patients, RBM45 co-localized with TDP-43 in cytosolic and rare intranuclear inclusions of dentate granule cells (arrows in Fig. 5d, e). We also observed co-localization of RBM45 to TDP-43 inclusions in the dentate of AD patients (Fig. 5f). A speckled RBM45 nuclear staining pattern was evident in many dentate granule cells that is distinct from the more diffuse TDP-43 nuclear immunostaining. Using multiple images from 10 ALS, 4 FTLD-TDP and 5 AD cases, we determined that RBM45 was present in 64 % of the TDP-43 inclusions in the spinal cord of ALS cases and 70 % of the TDP-43 inclusions in the hippocampus of FTLD-TDP and AD cases. Examples of TDP-43 inclusions that lack RBM45 are shown in Fig. 6. We observed ALS spinal cord motor neurons with nuclear RBM45 and weak or absent co-localization to TDP-43 cytoplasmic inclusions (Fig. 6a). In subjects with the *C9ORF72* repeat expansion, we also observed spinal cord motor neurons devoid of nuclear RBM45 and lacking RBM45 positive cytoplasmic inclusions (Fig. 6b). Finally, the hippocampus of ALS, FTLD-TDP and AD subjects contained TDP-43 inclusions without RBM45 (arrowheads in Fig. 6c). We did not detect RBM45 inclusions that completely lack TDP-43 immunoreactivity.

### Co-localization of RBM45 and ubiquitin

**Fig. 7:**
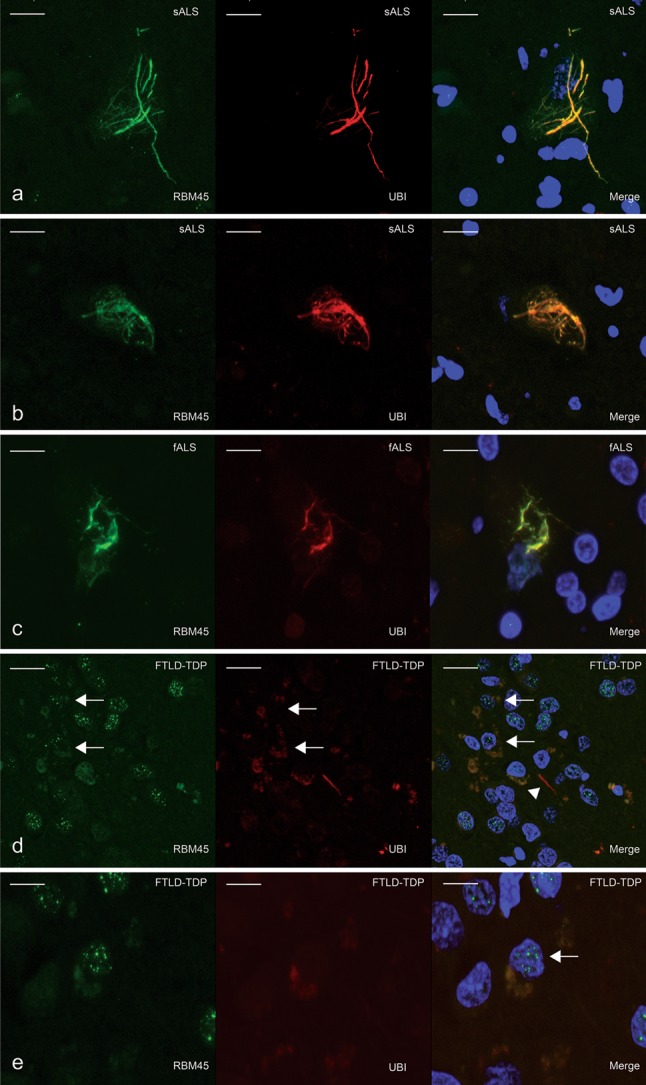
Double-label immunofluorescence for RBM45 (*green*) and ubiquitin (*red*) in the spinal cord and hippocampus. **a**–**c** RBM45 co-localization with ubiquitin in ALS spinal cord motor neurons. DAPI visualizes nuclei (*blue*) in the merged images. Cytoplasmic ubiquitin inclusions are positive for RBM45; however, nuclear RBM45 is not labeled by ubiquitin (**c**). **d**, **e** Co-localization of RBM45 to ubiquitin cytoplasmic inclusions in the dentate gyrus. *Arrows* denote cells that exhibit co-localization of RBM45 and ubiquitin within an inclusion but retaining a speckled RBM45 nuclear immunostain. Note the lack of RBM45 labeling of an ubiquitin positive dystrophic neurite in the dentate gyrus as indicated by *arrowhead* (**d**). *Scale bars* denote 20 μm in *panels* **a**–**d** and 30 μm in *panel* **e**. *Each panel* represents the following case numbers in Table 1: **a** 10, **b** 7, **c** 22, **d** 27, **e** 26

As ubiquitin-positive inclusions are a pathologic hallmark of both ALS and FTLD-TDP [14, 26], we examined the co-localization of RBM45 and ubiquitin-containing inclusions in ALS and FTLD-TDP. RBM45-containing cytoplasmic inclusions in lumbar spinal cord motor neurons of sporadic and familial ALS cases were frequently co-labeled with ubiquitin (Fig. 7a–c). RBM45 nuclear immunoreactivity in neurons or glia lacked ubiquitin staining (Fig. 7a, c). These results suggest that RBM45 pathology is highly coincident with ubiquitin in cytoplasmic inclusions, but nuclear RBM45 lacks ubiquitin.

Ubiquitin also labeled RBM45 cytoplasmic inclusions within dentate granule cells of FTLD-TDP patients (Fig. 7d, e). In addition, we also observed punctate RBM45 nuclear staining in cells that contained cytoplasmic ubiquitin-containing inclusions (Fig. 7d, e). However, we did not detect RBM45 within ubiquitin labeled dystrophic neurites in the dentate of FTLD-TDP subjects (Fig. 7d), and ubiquitin did not label the speckled nuclear RBM45 immunoreactivity (Fig. 7d, e).

## Discussion

We report a new RNA-binding protein that exhibits cytoplasmic inclusions in spinal motor neurons and dentate granule cells in ALS and FTLD-TDP patients. This protein, RBM45, was identified by an unbiased mass spectrometry-based proteomic screen of CSF and verified by immunoblot analysis. Additional RNA- and DNA-binding proteins were also observed in our CSF-based proteomics analyses that warrant further investigation (Table 4). We focused efforts in this study to RBM45 since it exhibited the most statistically significant *p* value and highest effect size between the ALS and control groups, was known to be developmentally regulated in the nervous system, contained structural similarities to TDP-43 and FUS, and commercial antibodies were available. RBM45 was detected in a punctate or speckled immunostaining pattern in the nucleus of neurons and glia. We observed RBM45-positive inclusions in motor neurons and glia in sporadic and familial ALS cases, as well as in hippocampal neurons of FTLD-TDP and AD patients. The most abundant RBM45 spinal cord pathology was detected in patients that harbor the *C9ORF72* hexanucleotide repeat expansion, with almost four times the density of motor neuron nuclear inclusions than ALS cases without the repeat expansion (12.4 vs. 3.4 inclusions/mm^2^). This was not due to increased numbers of remaining motor neurons in the *C9ORF72* cases but an increased percentage of the remaining motor neurons containing inclusions. There were no differences in the amount of glial RBM45 inclusions in *C9ORF72* repeat expansion cases versus ALS cases without the repeat expansion. We did not detect any intranuclear RBM45 inclusions in the spinal cord. Importantly, no RBM45 inclusions were detected in any region or cell type examined in control subjects. Significant co-localization of RBM45 with TDP-43 or ubiquitin was detected within inclusions of both ALS and FTLD-TDP patients, but RBM45 did not co-localize with tau pathology in AD cases. RBM45 within the nucleus was not labeled by ubiquitin and did not co-localize with TDP-43.

Few prior studies have investigated RBM45, and our study is the first to report on the distribution and expression of RBM45 in the human brain and spinal cord. In control subjects, we saw punctate staining of the nucleus and cytoplasm of motor neurons of the lumbar spinal cord, along with speckled nuclear staining of adjacent glia (Fig. 2a, b). This is consistent with the initial characterization of the protein in rodents, which suggested that the protein is capable of shuttling between the nucleus and cytoplasm [43]. Moreover, we detected limited, predominantly nuclear RBM45 in a speckled staining pattern within dentate granule cells of control subjects (Fig. 3a–c). Nuclear speckles were commonly observed in a variety of cell types, including neurons and glia. In mammalian cells, they occur in interchromatin regions of the nucleus and contain RNA [18, 40], and are dynamic structures most commonly observed using antibodies to splicing factors that function as regulators of pre-mRNA splicing [11, 29]. The speckled staining pattern seen for RBM45 is consistent with a role as an RNA-binding protein involved in pre-mRNA splicing.

The abundant level of RBM45 in the CSF of control subjects suggests that this RNA-binding protein may also have an extracellular function. Numerous RNA species are contained within biofluids and many have been reported as biomarker candidates for human diseases [6, 16], and while speculative, RNA-binding proteins such as RBM45 may modulate extracellular RNA signaling between cells. Recent evidence indicates that cultured cells stimulated to release microRNAs also release numerous RNA-binding proteins that may contribute to the protection of extracellular micoRNAs [45].

We established a connection between RBM45 and neurodegenerative disease by virtue of the cytoplasmic inclusion pathology observed in a total of 91 % of ALS, 100 % of FTLD-TDP and 75 % of AD cases. We did not identify any clinical attributes (age, gender, site of disease onset, disease duration) that would provide insight into why some ALS cases fail to exhibit RBM45 pathology. Further studies are necessary to explore the mechanisms of RBM45 inclusion formation and to characterize RBM45 in other neurodegenerative disorders. The pattern of RBM45 inclusion pathology strongly resembles that observed for TDP-43. Namely, we identified numerous cytoplasmic neuronal and glial inclusions in the lumbar spinal cord of sporadic and non-SOD1 familial ALS patients, as well as neuronal inclusions in the hippocampus in FTLD-TDP and AD patients. This pattern is similar to that seen for the related proteins TDP-43 and FUS in sporadic and non-SOD1 familial ALS cases [9, 23]. Indeed, we saw considerable overlap of RBM45 and TDP-43 pathology by confocal microscopy in ALS, FTLD-TDP and AD patients. However, we did not detect RBM45 in TDP-43 or ubiquitin-positive dystrophic neurites in the dentate gyrus of FTLD-TDP patients (Fig. 7). We also did not observe RBM45 in tau pathology in the hippocampus of AD patients (Fig. 4). Assessment of co-localization with FUS awaits future studies.

Overall, RBM45 co-localized with 64 % of cytoplasmic TDP-43 inclusions in ALS spinal cord and 70 % of TDP-43 inclusions in the hippocampus of FTLD-TDP and AD cases. By confocal microscopy we did not detect any RBM45 inclusions that were completely devoid of TDP43 immunoreactivity, suggesting that RBM45 inclusion formation is always associated with TDP-43 inclusions in these brain and spinal cord regions. By confocal microscopy we also noted a punctate or speckled RBM45 nuclear staining in neurons containing RBM45, TDP-43 or ubiquitin inclusions (Figs. 5, 6, 7). Retention of FUS in the nuclei of cells harboring cytoplasmic inclusions has been noted in ALS cases that do not harbor mutations in FUS [12]. TDP-43 inclusions may temporally occur earlier in the pathobiology of ALS and RBM45 may be later sequestered into these inclusions. Future cell culture studies are necessary to determine the temporal pattern of TDP-43 and RBM45 deposition within inclusions and if RBM45-containing inclusions are cytotoxic. Since many neurons were observed that had both cytoplasmic RBM45 and speckled nuclear RBM45, the formation of RBM45 inclusions does not preclude its normal distribution, and hence function, in the nucleus. It is possible that the loss of RBM45 from the nucleus is cytotoxic and future studies will explore this possibility.

Aside from RBM45’s affinity for poly(C) and poly (G) RNA [43], little is known about RBM45-mediated regulation of gene expression, and continued studies of RBM45 function in splicing may provide insight into disease-associated dysregulation of gene expression. Interestingly, the most abundant RBM45 pathology occurred in the spinal cord of subjects containing the *C9ORF72* repeat expansion. Recently, ubiquitin or p62 positive and TDP-43 negative inclusions were noted in non-motor regions including the hippocampus and cerebellum of subjects with the *C9ORF72* repeat expansion [1, 7, 34, 41], and it is possible that RBM45 may be contained within these inclusions in the cerebellum. We did not have access to cerebellar tissue in these cases to explore this question. We also noted reduced nuclear immunostaining for RBM45 in cases that harbor the *C9ORF72* repeat expansion, though studies using a larger number of subjects containing the repeat expansion is necessary to confirm this observation.

The presence of RBM45 pathology in a majority of ALS and all FTLD-TDP cases suggests that RBM45 inclusions occur via a pathway common to familial and non-familial forms of neurodegeneration. Prior studies have linked mutations or gene variations in a number of RNA processing proteins to ALS and FTLD-TDP, including TDP-43, FUS, senataxin, the survival motor neuron protein and ataxin-2 (reviewed in [2]). This raises the possibility that RBM45 mutations may also occur in familial forms of ALS or FTLD. However, the chromosomal location of the RBM45 gene, 2q31.2, has not been linked to ALS or FTLD in any prior genetic linkage study. Nevertheless, our data strengthen the link between ALS and FTLD-TDP to RNA metabolism (reviewed in [17, 42]). It is also important to note that TDP-43 has been shown to interact with the RNA for FUS and RBM45 in neurons, suggesting a functional role for TDP-43 in the expression of both FUS and RBM45 [39].

In summary, we have identified a novel RNA-binding protein in cytoplasmic inclusions of motor neurons and glia in sporadic and familial ALS, as well as in hippocampal neurons in FTLD-TDP and AD. RBM45 inclusions readily co-localize with both TDP-43 and ubiquitin, but RBM45 can be distinguished from these proteins through its speckled nuclear immunostaining and lack of incorporation into hippocampal dystrophic neurites. The most abundant RBM45 pathology typically occurred in patients that harbor the *C9ORF72* repeat expansion. Additional work is needed to understand how this protein contributes to both the normal RNA metabolism as well as to neurodegenerative disorders. Nevertheless, the identification of a new RNA-binding protein associated with these disorders holds promise for further mechanistic insights into disease pathogenesis and strengthens the role of RNA metabolism in these disorders [37, 47].

## Electronic supplementary material

Below is the link to the electronic supplementary material.

Supplemental Figure 1. Amino acid domain analysis of TDP-43, FUS, and RBM45. All have one or more RNA recognition motifs (RRMs). TDP-43 and FUS share a common glycine-rich-domain (GRD) that is absent from RBM45. A nuclear localization signal (NLS) is present in each protein, with a C-terminal location in both FUS and RBM45. RBM45 lacks a defined nuclear export signal (NES) present in both TDP-43 and FUS. FUS also contains a zinc finger-binding motif (ZnF). RBM45 amino acid domains were defined according to http://www.uniprot.org, and the location of each RRM is defined in the figure. (TIFF 4,555 kb)

Supplemental Figure 2. RBM45 pathology is recognized by multiple antibodies to RBM45. (**a**, **b**) Hippocampal RBM45 pathology is seen in adjacent sections of an AD case. RBM45 positive inclusions are marked with arrows. Antibodies used were HPA020448 generated to amino acids 1-50, AV41154 generated to amino acids 216-257, and PI462476 to amino acids 452-467. RBM45 inclusions are detected by affinity purified rabbit polyclonal anti-RBM45 antibodies HPA020448 and AV41154 as indicated by arrows in (**a**) and (**b**), respectively. Scale bar = 20 μm. (**c**, **d**). Multiple antibodies detect RBM45 pathology in lumbar spinal cord motor neurons in ALS cases. Adjacent sections of lumbar spinal cord from an ALS case were stained with anti-RBM45 antibodies HPA020448 (**c**) or affinity purified rabbit monoclonal antibody PI462476 (**d**). Inclusions are marked by arrows. Scale bar = 30 μm. (**e**, **f**) Anti-RBM45 antibody HPA020448 does not detect inclusions when pre-incubated with blocking peptide. Adjacent sections of a sALS case were incubated with anti-RBM45 antibody HPA020448 in the absence (**e**) or presence (**f**) of RBM45 blocking peptide (aa’s 1-50). In the absence of blocking peptide, the antibody detects RBM45 positive inclusions in motor neurons. When incubated with blocking peptide, however, all immunostaining is eliminated. An inclusion in (**e**) is marked with an arrow. Scale bar = 30 μm. Panels represent the following case numbers in Table 1: (**a** and **b**) = 30; (**c** and **d**) = 22; (**e** and **f**) = 10. (TIFF 12,088 kb)
