# Supplementary figures and images for: The RNA-binding motif 45 (RBM45) protein accumulates in inclusion bodies in amyotrophic lateral sclerosis (ALS) and frontotemporal lobar degeneration with TDP-43 inclusions (FTLD-TDP) patients
Source: Acta Neuropathol. 2012 Sep 21;124(5):717–32. doi: 10.1007/s00401-012-1045-x (PMC3472056; doi:10.1007/s00401-012-1045-x)

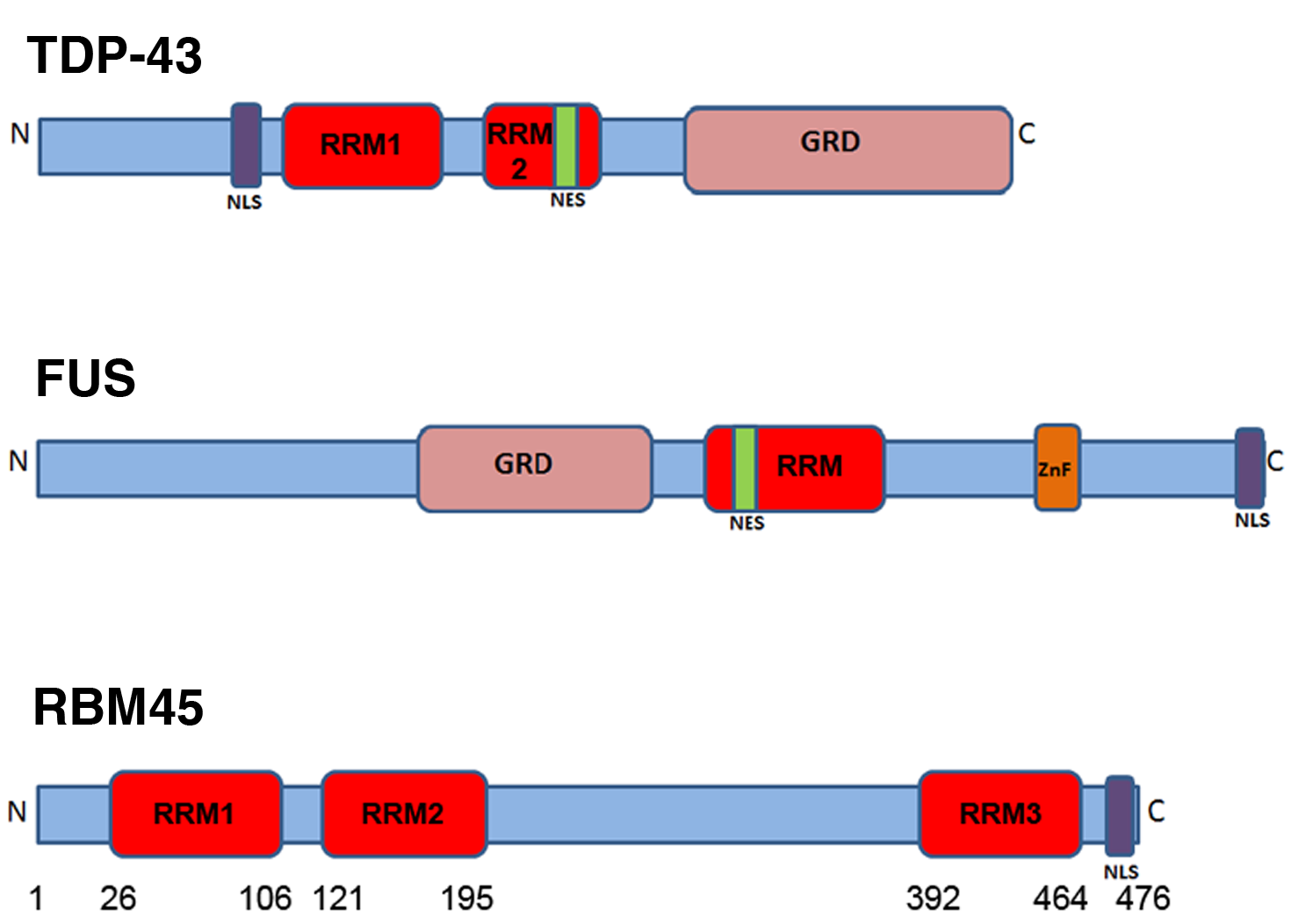

Supplement: Supplementary file 1 — Supplemental Figure 1. Amino acid domain analysis of TDP-43, FUS, and RBM45. All have one or more RNA recognition motifs (RRMs). TDP-43 and FUS share a common glycine-rich-domain (GRD) that is absent from RBM45. A nuclear localization signal (NLS) is present in each protein, with a C-terminal location in both FUS and RBM45. RBM45 lacks a defined nuclear export signal (NES) present in both TDP-43 and FUS. FUS also contains a zinc finger-binding motif (ZnF). RBM45 amino acid domains were defined according to http://www.uniprot.org, and the location of each RRM is defined in the figure. (TIFF 4,555 kb) [file 401_2012_1045_MOESM1_ESM.tif]

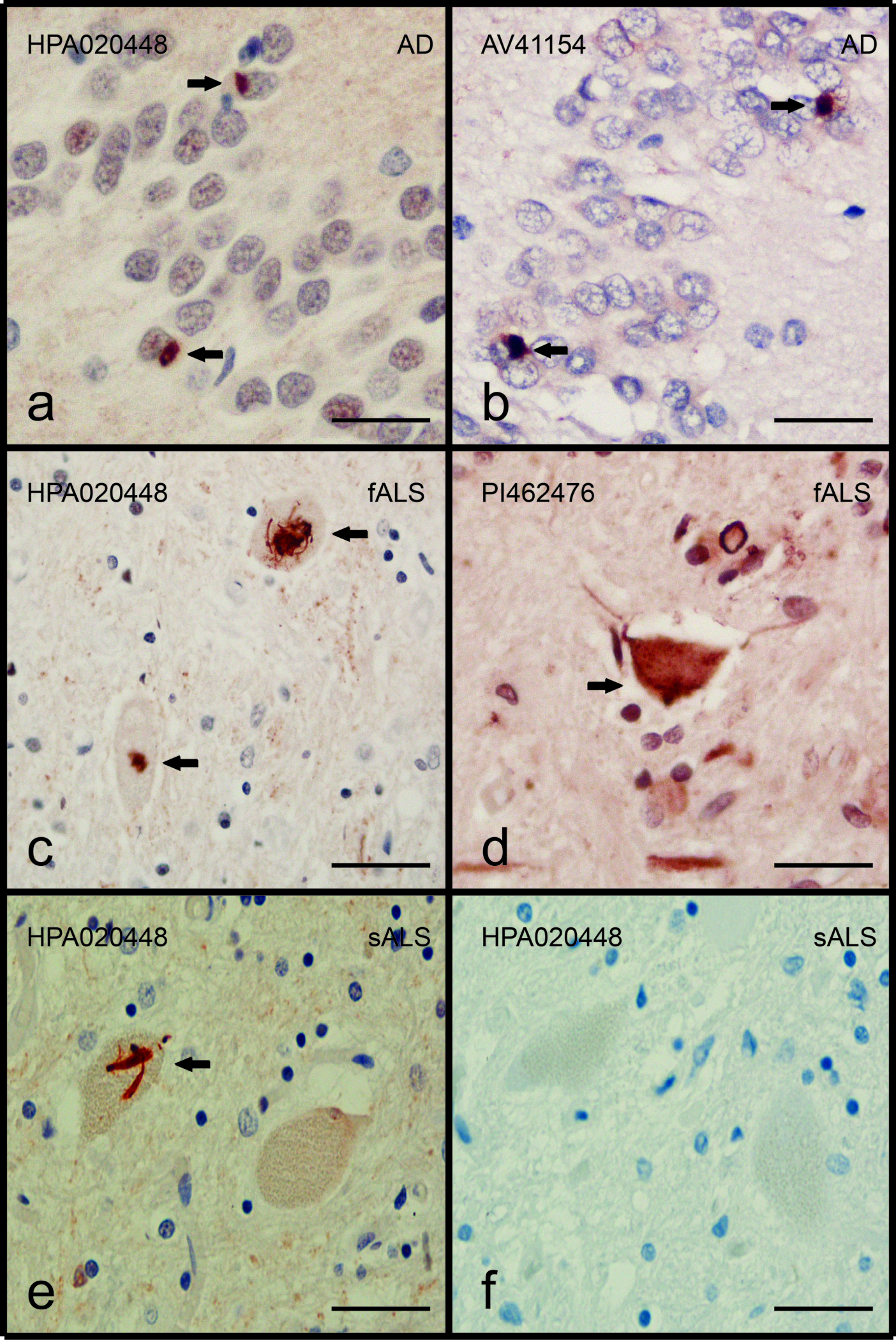

Supplement: Supplementary file 2 — Supplemental Figure 2. RBM45 pathology is recognized by multiple antibodies to RBM45. (a, b) Hippocampal RBM45 pathology is seen in adjacent sections of an AD case. RBM45 positive inclusions are marked with arrows. Antibodies used were HPA020448 generated to amino acids 1-50, AV41154 generated to amino acids 216-257, and PI462476 to amino acids 452-467. RBM45 inclusions are detected by affinity purified rabbit polyclonal anti-RBM45 antibodies HPA020448 and AV41154 as indicated by arrows in (a) and (b), respectively. Scale bar = 20 μm. (c, d). Multiple antibodies detect RBM45 pathology in lumbar spinal cord motor neurons in ALS cases. Adjacent sections of lumbar spinal cord from an ALS case were stained with anti-RBM45 antibodies HPA020448 (c) or affinity purified rabbit monoclonal antibody PI462476 (d). Inclusions are marked by arrows. Scale bar = 30 μm. (e, f) Anti-RBM45 antibody HPA020448 does not detect inclusions when pre-incubated with blocking peptide. Adjacent sections of a sALS case were incubated with anti-RBM45 antibody HPA020448 in the absence (e) or presence (f) of RBM45 blocking peptide (aa’s 1-50). In the absence of blocking peptide, the antibody detects RBM45 positive inclusions in motor neurons. When incubated with blocking peptide, however, all immunostaining is eliminated. An inclusion in (e) is marked with an arrow. Scale bar = 30 μm. Panels represent the following case numbers in Table 1: (a and b) = 30; (c and d) = 22; (e and f) = 10. (TIFF 12,088 kb) [file 401_2012_1045_MOESM2_ESM.tif]
